# Sometimes, the size matters: Wing geometric morphometrics as a tool to assess domiciliation by *Triatoma sordida* (Stäl 1859)

**DOI:** 10.1590/0037-8682-0516-2023

**Published:** 2024-08-09

**Authors:** Gilmar Ribeiro-Jr, Jamylle Reis, Fernanda Vaccarezza, Amanda Catariny de Oliveira Silva, Fernanda Cardoso Lanza, Diego Lopes Paim Miranda, Rodrigo Gurgel-Gonçalves, Mitermayer Galvão dos Reis

**Affiliations:** 1 Instituto Gonçalo Moniz, FIOCRUZ, Salvador, BA, Brasil.; 2 Universidade de Brasília, Faculdade de Medicina, Laboratório de Parasitologia Médica e Biologia de Vetores, Brasília, DF, Brasil.; 3 Universidade Federal da Bahia, Faculdade de Medicina, Salvador, BA, Brasil.; 4 Yale University, Connecticut, USA.

**Keywords:** Triatomines, Domiciliation, Morphometry

## Abstract

**Background:**

Domiciliation by *Triatoma sordida* is a public health concern in South America. This study aimed to evaluate the morphometric changes in the domestic and peridomestic populations of *T. sordida*.

**Methods:**

Specimen hemelytra were mounted, digitized, and processed for geometric morphometric analyses.

**Results:**

The specimens captured in houses were smaller than those captured in peridomiciles. A large size reduction effect was observed in female peridomicile populations compared with female house populations.

**Conclusions:**

*T. sordida* house populations were smaller than peridomestic populations. Wing geometric morphometry can be used as a tool to indicate *T. sordida* domiciliation.

Chagas disease (CD) is a neglected tropical disease that is transmitted by blood-sucking bugs of the Triatominae subfamily, popularly known as kissing bugs. Owing to their ability to colonize houses, some triatomine species are closely associated with *Trypanosoma cruzi* transmission to humans and domestic animals^1^. Understanding the determinants of triatomine invasion, infestation, and house colonization provides relevant information for CD surveillance^2^. 

*Triatoma sordida* has a wide geographic distribution, is commonly found in wild environments infected with *T. cruzi*, and is the most frequent synanthropic triatomine species in Brazil, where it is strongly associated with chicken coops^1^. Despite its low competence to transmit *T. cruzi* due to its ornithophilic habits, *T. sordida* specimens can disperse up to 32 m in peridomestic rural areas^3^ ^,^ ^4^ and reach houses, maintaining the risk of CD transmission^5^. Thus, mapping the areas of *T. sordida* house infestations is extremely relevant for guiding CD prevention strategies.

Variations in the size, shape, and color of triatomines have been described as adaptive responses to different microhabitats^6^. Other studies have described a decrease in body size and sexual dimorphism as relevant markers of triatomine domiciliation^7^ ^-^ ^9^. This evidence suggests that peridomestic triatomine populations can be differentiated from domestic populations, with size differences indicating an old house infestation. Geometric morphometry is widely used for evaluating triatomine populations. This approach uses anatomical landmarks and image superposition methods to analyze variations in size and shape between individuals. This technique was previously applied to differentiate *T. sordida* from similar species^9^ ^-^ ^11^ and to analyze the morphometric variation of *T. sordida* in Brazil^11^. As size changes are associated with house infestation, we aimed to evaluate the existence of morphometric modifications in domestic and peridomestic populations of *T. sordida*. We hypothesized that 1) the wing size of *T. sordida* populations captured inside houses is smaller than that of peridomestic populations and 2) sexual size dimorphism is smaller among domestic populations.

Triatomine samples were collected from January 2013 to December 2014 from six localities in the municipality of Barra, Bahia (Figure 1A), according to previously described methods^12^. Triatomines were identified using dichotomous identification keys in a double-verification process. The insects were marked with numbers and QR codes using Zebra® TLP 2844 printer and Zebradesign® V.2 software. All samples were deposited in the entomological collection of Fiocruz Bahia. Triatomine wing samples (hemelytra) were selected for geometric morphometric analysis owing to the method’s feasibility and reproducibility. The hemelytra were placed between glass microscope slides, fixed with a double-sided tape, and scanned at a high resolution (900 dpi). The images were edited using PhotoScape software and organized according to sex and collection environment. We digitized seven anatomical points on each wing (Type I and II) using TpsDig® software^9^ to determine the coordinates (x and y). The wing size was obtained using an estimator of size (centroid size) and calculated using the coordinates of each wing anatomical point using TPSrelw® software, which also performed wing coordinate alignment and shape variable determination.

**FIGURE 1:**
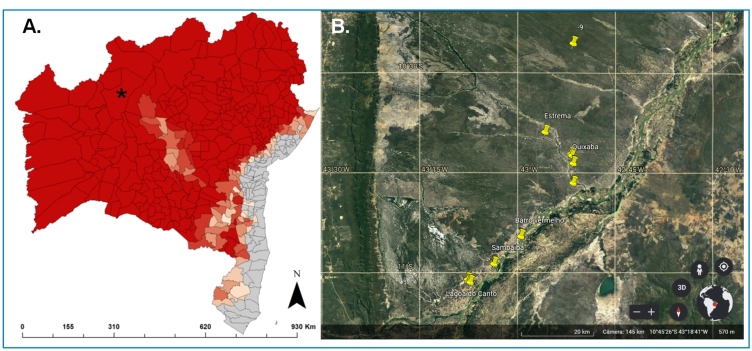
Study area. **(A)** *Triatoma sordida* spatial distribution in Bahia state, *showing the municipality of Barra. **(B)** Google Earth satellite imagery of household units where triatomines were captured. The yellow markers show the sampling locations of *Triatoma sordida* between 2013 and 2014; São Francisco River margin locations: Estrema, Quixaba, Barro Vermelho, Sambaíba, and Lagoa do Canto; −9 = non-informed location.

A factorial analysis of variance (ANOVA) was carried out to determine the effects of sex and habitat (intradomestic or peridomestic) on the size of *T. sordida* specimens. Shape variables derived from the TPSrelw partial deformations were used for the principal component analysis (PCA). A factorial map of the first two principal components was created to illustrate the main results. The results of ANOVA and PCA were calculated using Statistica® software (StatSoft, Inc., Tulsa, OK, USA).

We captured 410 *T. sordida* specimens in Barra City (Figure 1B), found in domiciles (53.9%, n=221) and peridomiciles (46.1%, n=189), among 10 localities and 33 colonies, with an average of 12 (standard deviation=7.6) triatomines per colony (Table 1). All peridomestic colonies were found in chicken coops, while domestic colonies were found in kitchens, bedrooms, and living rooms. The wing geometric morphometric analysis of 120 adults, 63 from peridomiciles and 57 from houses (Table 1), revealed that *T. sordida* females captured inside houses were much smaller than those captured in peridomiciles, while male specimens showed similar sizes between the environments. As expected, sexual dimorphism was observed, with female specimens being larger than male specimens in peridomiciles. However, we did not find any size differences between males and females from the houses. Therefore, the size decrease effect between habitats (ANOVA F1.116 = 5.86, p=0.017) was more pronounced for female specimens (Figure 2A) and that size sexual dimorphism was lower in domestic populations of *T. sordida*. The PCA did not reveal marked wing shape differences between the domestic and peridomestic populations (Figure 2B).

**TABLE 1:** Information about the colonization of houses and peridomestic environments by *T. sordida* in the municipality of Barra, Bahia state, by assessed location.

Locality	Houses				Peridomicile				Total
	♀	♂	Nymph	Subtotal	♀	♂	Nymph	Subtotal	
Barro Vermelho	18	12	13	53 (14)					53
Lagoa do canto	26	45	34	105 (24)					105
Sambaíba	18	26	18	62 (19)					62
Boca do saco					8	8	5	21	21
Brejinho					14	10	20	44 (19)	44
Extrema					15	16	15	46 (11)	46
Quixaba					18	16	12	46 (33)	46
−9 (non-informed)					14	6	13	33	33
**Total**	62	83	75	**220 (57**)	69	56	65	**190 (63**)	**410**

**Legend:** Values in the parentheses ( ) represent the samples selected for geometric morphometry analysis.

**FIGURE 2:**
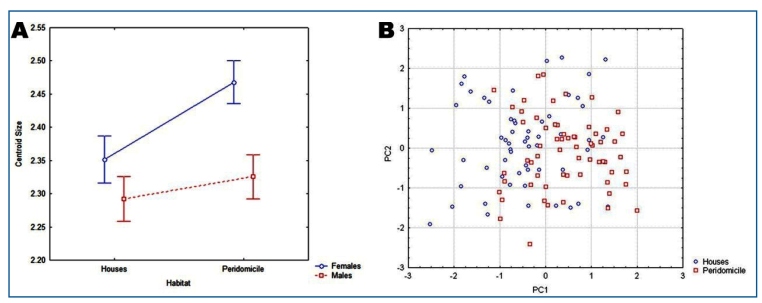
**(A)** Factorial analysis of variance of the centroid size of male and female specimens of *Triatoma sordida* according to the collecting habitat. **(B)** Principal component analysis of the *Triatoma sordida* specimens’ adult wing shape collected from the intra- and peridomestic environments in the municipality of Barra, Bahia state.

Triatomine specimens captured inside houses were smaller than those captured in the peridomestic areas. In addition, the size difference between sexes decreased, mainly due to the smaller size of *T. sordida* female specimens captured in houses. Geometric morphometric analyses were conducted to assess any intraspecific variations^10^ and to determine whether local reinfestations originated from wild or synanthropic populations with residual foci^13^. 

As expected, smaller populations were found in houses, which agrees with the results found in other species, such as *T. infestans*. Furthermore, the population size differences between environments were more remarkable among female specimens, and sexual dimorphism was more pronounced in the peridomestic population. Dujardin et al.^13^ demonstrated similar results when the wild and laboratory populations of *T. infestans* and *Rhodnius domesticus* were compared. They noted that the changes occurring during the transition from natural to artificial environments influence the amount and quality of food for triatomines, which is related to size the differences between male and female specimens. Dujardin et al.^13^ recommended the use of size-based sexual dimorphism as an essential tool for examining triatomine adaptation in domestic environments. Our results support the use of sexual dimorphism analysis to determine the processes of adaptation to houses and confirm the hypothesis that *T. sordida* has adapted to colonize houses in Barra City, Bahia.

The process of house colonization by *T. sordida* can be affected by inadequate management of poultry, especially domestic chickens (*Gallus gallus domesticus* L.)^14^. Additionally, a strong association between *T. sordida* and poultry has been well-documented, explaining its high incidence in peridomestic chicken coops^15^. The house colonization of *T. sordida* can be attributed to its ability to move around the peridomestic environment^3^ ^,^ ^4^ and is facilitated by the local human behavior of raising birds and keeping bird nests inside household units, especially for laying eggs. Thus, *T. sordida* specimens could enter household units either passively (as small nymphs) or actively (as nymphs and adult triatomines walking and being actively attracted by poultry) enter household unit^4^.

The colonization of artificial anthropic environments by *T. sordida* has been documented, demonstrating a progressive adaptation of the species across the national territory, including the Bahia state^12^. Between January 2013 and December 2014, 5,906 triatomines of 15 species were captured from 127 municipalities across all regions of Bahia. Approximately 3,500 specimens (60%) were *T. sordida* species, infesting 70 (55%) of the sampled municipalities^12^. Therefore, it is important to investigate the factors influencing *T. sordida* domiciliation. The use of geometric morphometry as a surveillance tool to access domiciliation is feasible, low cost, and reproducible. This tool offers benefits such as the possibility of comparing information among different areas, strengthening the entomological surveillance system.

In addition to geometric morphometrics, genetic analyses can be used to study triatomine domiciliation. Monteiro et al.^16^ compared the genetic structure of intradomestic and peridomestic *T. sordida* populations in Brazil and found low genetic variation among the peridomestic populations. The relevance of these studies is based on the current evidence for domiciliation described in our study.

New tools may strengthen surveillance of vector-borne CD. Two interesting perspectives for further characterization of domestic and peridomestic *T. sordida* populations in this area would be (1) to characterize the populations using microsatellites and (2) to analyze the head morphometry of nymphs found inside and outside houses. We also recommend further research in other areas of Bahia with a history of domestic (intra- and peridomestic) colonization by *T. sordida* to confirm the process of domestic colonization by this species, thus reinforcing its role as a potential *T. cruzi* vector*.* Finally, our results suggest the use of wing geometric morphometry as a tool to indicate *T. sordida* domiciliation; hence, we believe that the use of this tool could improve vector-borne CD surveillance. 

## ● Ethics

The procedures were performed in accordance with the ethical standards of the Research Ethics Committee of the Gonçalo Moniz Institute (IGM; FIOCRUZ, Bahia, Brazil). The requirement for obtaining informed consent was waived as the analysis was performed using the Bahia Entomological Surveillance System samples. However, no personal identification data were used to ensure complete anonymity of the participants. The research did not cause any physical, psychological, moral, intellectual, social, cultural, or religious risks to the residents or animals in the study area. Additionally, this study did not include endangered or protected species.
